# Printed 5-V organic operational amplifiers for various signal processing

**DOI:** 10.1038/s41598-018-27205-7

**Published:** 2018-06-12

**Authors:** Hiroyuki Matsui, Kazuma Hayasaka, Yasunori Takeda, Rei Shiwaku, Jimin Kwon, Shizuo Tokito

**Affiliations:** 1 Research Center for Organic Electronics (ROEL), Yamagata University, Yonezawa, 992-8510 Japan; 2 Department of Creative IT Engineering, Pohang University of Science and Technology (POSTECH), Pohang, 37673 Korea

## Abstract

The important concept of printable functional materials is about to cause a paradigm shift that we will be able to fabricate electronic devices by printing methods in air at room temperature. One of the promising applications of the printed electronics is a disposable electronic patch sensing system which can monitor the health conditions without any restraint. Operational amplifiers (OPAs) are an essential component for such sensing system, since an OPA enables a wide variety of signal processing. Here we demonstrate printed OPAs based on complementary organic semiconductor technology. They can be operated with a standard safe power source of 5 V with a minimal power consumption of 150 nW, and used as amplifiers, a variety of mathematical operators, signal converters, and oscillators. The printed micropower organic OPAs with the low voltage operation and the high versatility will open up the disposable electronic patch sensing system in near future.

## Introduction

Printed electronics is an emerging field where electronic devices are manufactured by printing technologies such as inkjet printing and gravure offset printing with electronic functional inks instead of color inks^1–3^. Printing technologies do not require vacuum processes and enable direct patterning without photolithography, which are believed to reduce the manufacturing cost significantly. In particular, the printed electronics based on organic semiconductors can be fabricated typically below 150 °C, while printed oxide semiconductors require the temperatures above 230 °C to get their inherent performance^4^. Flexible displays, electronic papers and large-area sensor sheets, where organic thin-film transistors (OTFTs) are used for the active matrices, have been considered as the promising applications of the printed organic electronics for a long time^5–7^. More recently, the expectations for other applications such as wireless tags and bio-sensors are increasing considerably^8–11^. This is because printable and air-stable p-type and n-type organic semiconductor materials have been developed, and enabled the fabrication of complementary digital and analog circuits^12,13^. In both wireless tags and bio-sensors, printed analog circuits are essential since the transmitting radiowaves and the outputs of a variety of sensors are in most cases analog signal.

Operational amplifiers (OPAs) are one of the most useful elements for analog processing, particularly used in the silicon technology, because of their exceptional versatility^14^. As the name suggests, OPAs are not just amplifiers but can be used for a variety of operations including addition, subtraction, integration and differentiation. Furthermore, they can be used for a variety of signal processing such as active filters (low-pass, high-pass, etc.), impedance converters, current-to-voltage converters, oscillators and comparators. So far, several printed or vacuum-processed organic OPAs have been reported as summarized in Table 1 ^15–19^. However, the reported printed organic OPAs have required power sources of more than 40 V, which are not usually available in mobile electronic devices and are not safe in bio-applications. Power sources of 5 V are standard for mobile electronics, since they are safe, can be obtained directly from a small battery without bulky DC-DC converters, and can be shared with the power source for microcontrollers. In addition, the versatility, one of the most important nature of OPAs, of the printed organic OPAs has never been demonstrated.

**Table 1:** Comparison of this study and related studies on organic operational amplifiers (OPAs).

Year	Fabrication Method	Supply Voltage (V)	Power Consumption (µW)	Gain Bandwidth Product (Hz)	Reference
2018	Printing	5	0.15	50	This work
2013	Printing	50	325	75	^15^
2011	Printing	40	40	n.a.	^16^
2012	Photolithography	15	225	2000	^17^
2011	Photolithography	15	315	500	^18^
2011	Photolithography	5	2.75 × 10^−4^	7.5	^19^

Here we report on the printed organic OPAs operatable at a standard supply voltage of 5 V and demonstrate their versatility for a variety of signal processing. The organic OPAs were fabricated as complementary circuits using solution-processed p-type and n-type OTFTs. The p-type and n-type semiconductors were blended, respectively, with polymer insulators in solution to reduce the device variability^20,21^. All metallic electrodes and interconnections were fabricated by inkjet printing, which is an ideal printing method because of digital on-demand process, from a silver nanoparticle ink. The dual-gate structure was employed for n-type OTFTs to make the switching performance more suitable for amplifiers. The fabricated organic OPAs could be operated at a supply voltage of 5 V at a power consumption of less than 150 nW, and exhibited an open-loop gain of 60 at maximum. The organic OPAs were then packaged into a dual inline package (DIP) for easier prototyping. Using various negative feedbacks with resistors and capacitors, the organic OPAs finally realized the gain-controllable amplifiers, integrators, differentiators, current-to-voltage converters, pulse-width-modulated oscillators and comparators.

## Results and Discussion

### P-type and n-type organic thin-film transistors (OTFTs)

**Figure 1:**
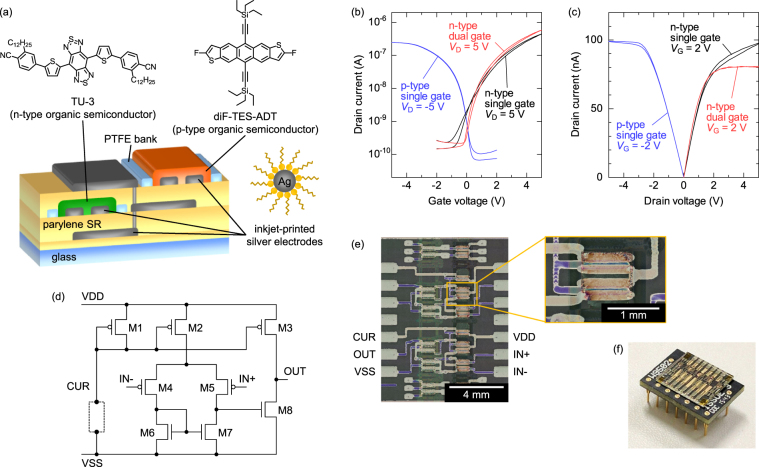
Device structure, photographs and transistor characteristics. (**a**) Schematics of the device structure. (**b**) Transfer and (**c**) output characteristics of p- and n-type organic transistors. (**d**) Circuit diagram of the organic operational amplifiers (OPAs). CUR terminal was connected to VSS via an external current source or a resistor. (**e**) Optical microscope images of the organic OPAs. (**f**) Photograph of the organic OPAs mounted on a dual inline package (DIP).

P-type and n-type OTFTs were fabricated on glass substrates in the structure illustrated in Fig. 1a. All layers except for gate insulators were fabricated by solution processes. An air-stable and soluble n-type semiconductor, 4,8-bis[5-(3-cyanophenyl)thiophene-2-yl]benzo[1,2-*c*:4,5-*c*’]bis[1,2,5]thiadiazole derivative (TU-3), was blended with poly-α-methylstyrene (PαMS) to reduce the device variability^13^. An air-stable and soluble p-type semiconductor, 2,8-difluoro-5,11-bis(triethylsilylethynyl)anthradithiophene (diF-TES-ADT), which possess a similar mobility as TU-3, was blended with polystyrene (PS) for the same reason as PαMS^22^. Fig. 1b,c show the transfer and output characteristics of the p-type and n-type OTFTs. The single-gate p-type OTFTs exhibited a hole mobility of *µ* = 0.08 ± 0.01 cm^2^ V^−1^ s^−1^ and a threshold voltage of *V*_th_ = 0.35 ± 0.09 V in the saturation regime for ten devices and a turn-on voltage *V*_ON_ close to zero. For high-gain amplifiers, the following two parameters are more important: a subthreshold slope, $SS\equiv \partial {V}_{{\rm{G}}}/\partial (\mathrm{log}\,{I}_{{\rm{D}}})$, giving the steepness of the switching of drain current^23^, and a channel length modulation coefficient, $\lambda \equiv (\partial {I}_{{\rm{D}}}/\partial {V}_{{\rm{D}}})/{I}_{{\rm{D}}}$, giving the degree of current saturation at high drain voltage^24^. Here *V*_G_ is the gate voltage, *I*_D_ is the drain current, and *V*_D_ is the drain voltage. Note that, although the origin of the drain voltage dependence of saturation current in OTFTs might be different from that of the channel length modulation effect in silicon MOSFETs, we use the same terminology for convenience. The p-type OTFTs exhibited *SS* = 0.3 V dec^−1^ and *λ* = 0.016 V^−1^. On the other hand, the single-gate (top-gate) n-type OTFTs exhibited an electron mobility of µ = 0.06 ± 0.02 cm^2^ V^−1^ s^−1^ and a threshold voltage of *V*_th_ = 0.10 ± 0.07 V for ten devices, a subthreshold slope of *SS* = 1 V dec^*−*1^, and a channel length modulation coefficient of *λ* = 0.04 V^−1^. As we will discuss in the following section, the *SS* and *λ* larger than those of p-type OTFTs degrade the performance of the complementary organic OPAs considerably. In order to improve the *SS* and *λ* for the n-type OTFTs, we fabricated dual-gate n-type OTFTs which have gate electrodes both at top and bottom sides of the semiconductor layer^25^. As a result of the dual-gate structure, the *SS* and *λ* of the n-type OTFTs were improved to 0.4 V dec^−1^ and 0.005 V^−1^, respectively, with a small decrease of a mobility to 0.04 ± 0.01 cm^2^ V^−1^ s^−1^ for ten devices. Transfer characteristics of all OTFTs were shown in Supplementary Figure S1. Device simulations have revealed that the improvements of the *SS* and *λ* were due to the suppression of the current flow at the bottom side of the channel layer, which could not be well controlled by top gate only (see Supplementary Information). The two types of OTFTs exhibited balanced mobilities suitable to complimentary circuits. Furthermore, the on/off ratios higher than 10^3^ in the voltage range between −5 V and 0 V for p-type and between 0 V and +5 V for n-type indicate that the complementary circuits can operate at a supply voltage of 5 V.

### Open-loop DC characteristics of the organic operational amplifiers (OPAs)

Using the single-gate p-type OTFTs and the dual-gate n-type OTFTs, we fabricated two-stage organic OPAs illustrated in Fig. 1d ^14^. VDD, VSS, IN+, IN−, OUT, and CUR denote positive voltage supply, negative voltage supply, non-inverting input, inverting input, output, and current source terminals, respectively. VDD and VSS were kept at +2.5 V and −2.5 V in all measurements, meaning that the supply voltage is totally 5 V. CUR should be connected to a current source or a resistor whose another end is connected to VSS. The three p-type OTFTs at the top (M1-M3) function as current sources with the same current value, composing so called current mirror. The input stage (M2, M4-M7) was used to amplify the input difference, *V*_IN+_ − *V*_IN−_, and convert it to a single-ended signal, while the common-mode signal, (*V*_IN+_ + *V*_IN−_)/2, is rejected. The gain stage (M3, M8) was used to provide a high voltage gain. As a result, the output voltage of an ideal operational amplifier can be expressed as

1 $${V}_{{\rm{OUT}}}={A}_{{\rm{open}}}({V}_{{\rm{IN}}+}-{V}_{{\rm{IN}}-}),$$

where *A*_OPEN_ is the open-loop gain. The gain stage may be followed by an additional stage called output stage to reduce the output impedance if necessary. Figure 1e shows the optical microscope image of the fabricated organic OPAs. The linewidth of metal interconnections was 200 µm for horizontal lines and 100 µm for vertical lines. The channel length was 16–32 µm, and channel width was 1000 µm. The area of the printed organic OPA was 4.3 × 4.4 mm^2^. For prototyping, the printed organic OPAs were mounted on a dual inline package (DIP) as shown in Fig. 1f.

**Figure 2:**
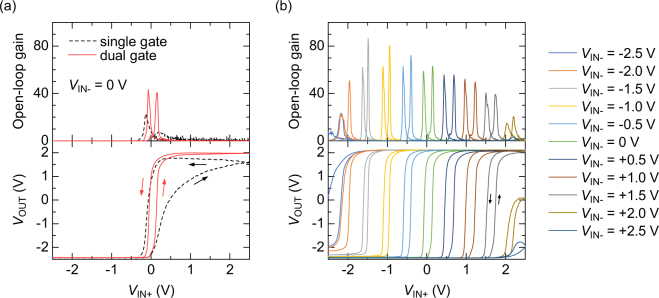
Open-loop DC characteristics of the organic OPAs. (**a**) Comparison of two types of organic OPAs with single-gate and dual-gate n-type OFETs. The geometry of p-type OFETs is single gate for both OPAs. (**b**) Organic OPA characteristics at various *V*_IN−_ voltages.

The DC characteristics of the organic OPAs were evaluated at first without any feedback, that is, open-loop condition. Fig. 2a compares the characteristics of the two organic OPAs with single-gate and dual-gate n-type OTFTs (see Supplementary Figure S2 for a larger number of OPAs). The CUR terminal was connected to a current source of 20 nA. As *V*_IN+_ was swept with keeping *V*_IN−_ at 0 V, the dual-gate organic OPAs exhibited a steep change of the output voltage, while the single-gate organic OPAs exhibited slow and hysteretic characteristics. Thus, the dual-gate structure for n-type OTFTs was found quite beneficial to get a high gain and small hysteresis in organic OPAs. The switching voltage in the dual-gate OPA was + 0.15 V for forward sweep and −0.04 V for backward sweep. Figure 2b shows the dependence of *V*_OUT_ on *V*_IN+_ in the dual-gate organic OPAs at fixed *V*_IN-_ of −2.5, −2.0, …, +2.5 V. The results indicate that the dual-gate organic OPAs have open-loop gains higher than 40 in a wide range of input voltages from −2.0 to +1.5 V. These characteristics can also be used as a comparator, which outputs high voltage when *V*_IN+_ > *V*_IN−_, and low voltage when *V*_IN+_  < *V*_IN−_.

### Closed-loop DC characteristics of the organic OPAs

**Figure 3:**
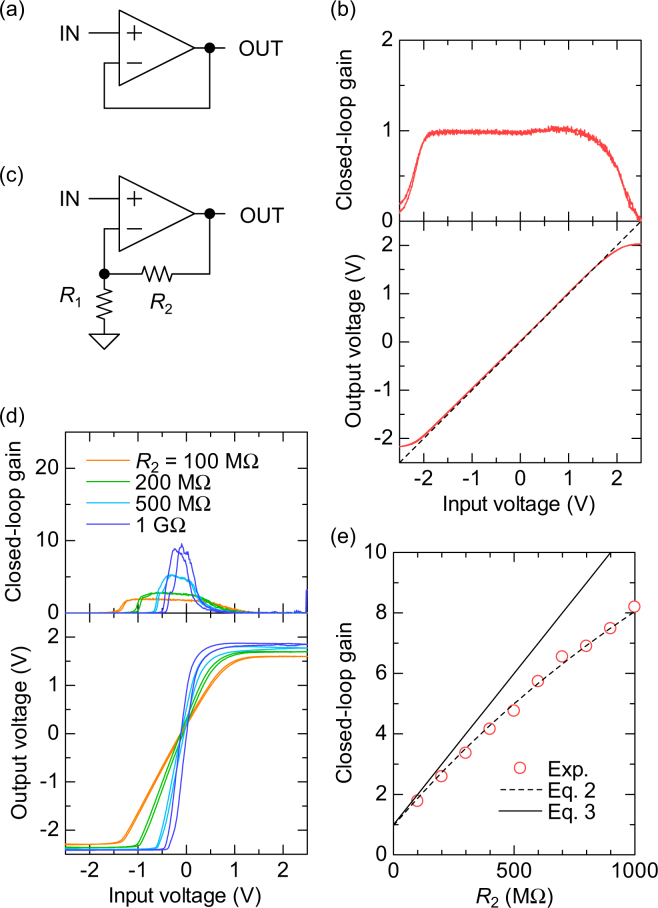
Closed-loop DC characteristics of the organic OPAs. (**a**) The circuit diagram and (**b**) the characteristics of the voltage follower. (**c**) The circuit diagram and (**d**) the characteristics of the non-inverting amplifier. The resistance *R*_1_ was fixed at 100 MΩ. (**e**) The dependence of closed-loop gain on *R*_2_ for the non-inverting amplifiers. Dashed line indicates the calculation based on Eq. 2 with an open-loop gain of 30.

The most important nature of OPAs can be seen when the OUT terminal is connected to the IN- terminal via passive elements such as resistors and capacitors. The connection gives a negative feedback, and this condition is called closed loop. The simplest closed-loop circuit is a voltage follower shown in Fig. 3a, which outputs the voltage same as the input. Figure 3b shows the characteristics of the voltage follower. The offset voltage |*V*_OUT_ − *V*_IN+_| was found less than 0.05 V, and the closed-loop gain was 0.99 ± 0.01. Hysteresis was significantly reduced from 0.19 V in the open loop to 0.005 V in the voltage follower by the negative feedback. Such voltage followers can be used for transmitting the signals from high-impedance sensors.

Another popular closed-loop circuit is a non-inverting amplifier shown in Fig. 3c, which amplify a voltage signal by a tunable gain. Based on Eq. 1, the output voltage of the non-inverting amplifier can be given by

2 $${V}_{{\rm{OUT}}}=\frac{1}{\frac{{R}_{1}}{{R}_{1}+{R}_{2}}+\frac{1}{{A}_{{\rm{open}}}}}{V}_{\mathrm{IN}+}\equiv {A}_{{\rm{closed}}}{V}_{\mathrm{IN}+}$$

where *A*_closed_ is a closed-loop gain. If the open-loop gain is high enough, ${A}_{{\rm{open}}}\gg ({R}_{1}+{R}_{2})/{R}_{1}$, the closed-loop gain is given simply by

3 $${A}_{{\rm{closed}}}=\frac{{R}_{1}+{R}_{2}}{{R}_{1}}.$$

Thus, the gain of the non-inverting amplifiers can be easily controlled by the ratio of *R*_1_ to *R*_2_. The controllability of the closed-loop gain in OPAs is in sharp contrast to the gain of common-source amplifiers with a resistor load. The gain of common-source amplifiers is given by $-R\mu C({V}_{{\rm{in}}}-{V}_{{\rm{th}}})W/L$ and is affected by a variety of parameters: the load resistance *R*, the mobility *µ*, the capacitance per unit area *C*, the input voltage *V*_in_, the threshold voltage *V*_th_, the channel width *W* and the channel length *L*^26^. Since variation exists in each parameter, the fine control of the gain in common-source amplifiers is quite difficult. DC characteristics of the organic non-inverting amplifiers is shown in Fig. 3d,e. When *R*_1_ = *R*_2_ = 100 MΩ (orange line), the closed-loop gain was 1.8, which is roughly consistent with the Eq. 3. As *R*_2_ was increased up to 1000 MΩ, the closed-loop gain increased up to 8.2. The obtained closed-loop gain was quite consistent with Eq. 2, while it shows the deviation of 25% at maximum from the Eq. 3. Increasing the open-loop gain *A*_open_ can reduce the deviation further.

### Frequency characteristics of the organic OPAs

**Figure 4:**
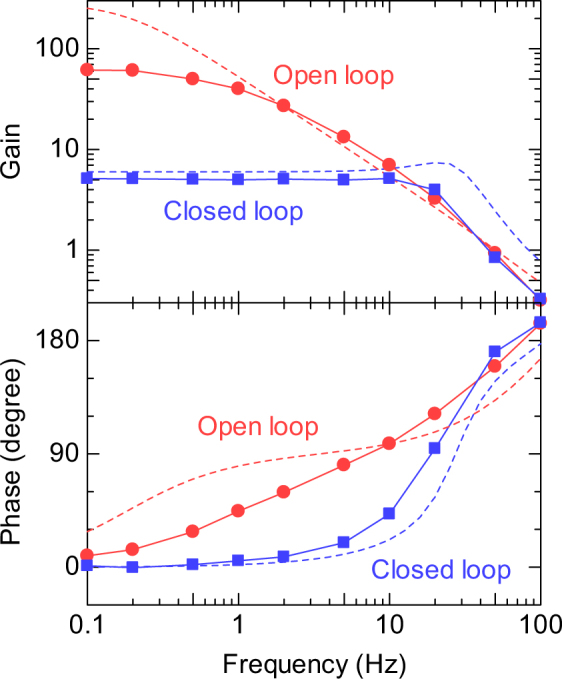
Frequency characteristics of the organic OPAs. Frequency dependence of the small-signal gain and phase for the open-loop organic OPA and the closed-loop non-inverting amplifier. *R*_1_ = 100 MΩ and *R*_2_ = 500 MΩ was used for the non-inverting amplifier. Dashed lines indicate the simulation curve by using the LTspice software.

To evaluate the dynamic characteristics of the organic OPAs, the frequency dependence of the open-loop gain and phase for the input of sine wave was measured between 0.1 and 100 Hz (Fig. 4). Hereafter, the CUR terminal was connected to VSS via a resistor of 100 MΩ, which approximately defines the current through the transistor M1 as 50 nA. At 0.1 Hz, the open-loop gain was 60 and the phase was close to zero. As the frequency increased, the open-loop gain decreased and the phase deviated from zero. The gain bandwidth (GB) product, defined by the frequency where the gain is unity, was estimated at 50 Hz, which is close to that of the reported one^15^. Note that our organic OPAs were operated at 5 V, whereas the reported ones were operated at 40 V. Since the cut-off frequency of the transistors is usually proportional to the supply voltage, *f*_c_ = *µV*/2π*L*(*L* + Δ*L*), the GB product is expected to increase at higher supply voltage. Further improvements of the GB product can be expected if we can reduce the channel length *L* and overlap length Δ*L*, for example, by finer printing methods such as reverse-offset printing^27^.

The negative feedback of the non-inverting amplifiers can again help to control the gain in a wide frequency range. At *R*_1_ = 100 MΩ and *R*_2_ = 500 MΩ, for example, the closed-loop gain was kept at 5 in the frequency range of 0.1–10 Hz. The phase could be also kept close to zero in the same frequency range. The controllable and frequency-independent gain is one of the advantages of the OPAs over the common-source amplifiers.

### Various signal processing by the organic OPAs

**Figure 5:**
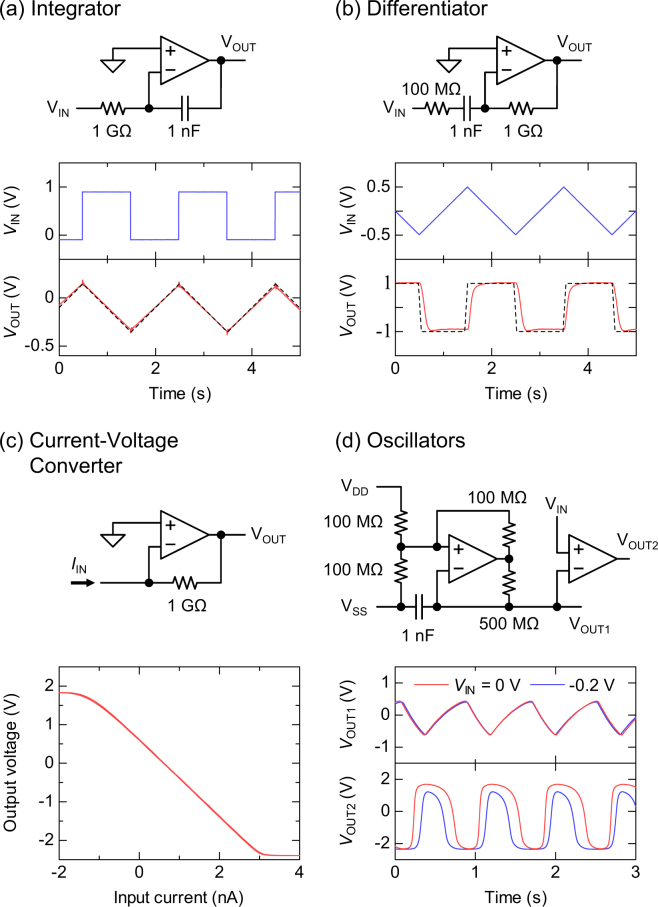
A variety of signal processing by the organic OPAs. (**a**) Integrator. (**b**) Differentiator. (**c**) Current-to-voltage converter (transimpedance amplifier). (**d**) Triangular oscillator and pulse width modulator.

Finally, we demonstrate that the printed organic OPAs can be utilized for a variety of signal processing. Connecting one resistor and one capacitor to the organic OPA, an integrator was fabricated as shown in Fig. 5a. As a result of the input of a rectangular wave, the integrator successfully output the triangular wave, which is the integration of the rectangular wave. The experimental data (solid line) is quite consistent with the theoretical curve (dashed line), ${V}_{{\rm{OUT}}}(t)=-{(RC)}^{-1}{\int }_{0}^{t}{V}_{{\rm{IN}}}(t){\rm{d}}t$, in the figure. The second demonstration is a differentiator, which was fabricated with one organic OPA, two resistors and one capacitor (Fig. 5b). The fabricated differentiator successfully differentiated the triangular wave into the rectangular wave. The experimental data (solid line) is quite consistent with the theoretical curve (dashed line), ${V}_{{\rm{OUT}}}(t)=-\,RC({\rm{d}}{V}_{{\rm{IN}}}(t)/{\rm{d}}t)$. The third demonstration is the current-to-voltage converter, or the transimpedance amplifier, which can be used, e.g., for the amperometric bio-sensors^28^. Figure 5c shows the circuit diagram and the input-output characteristics of the current-to-voltage converter. By using a 1 GΩ resistor as a feedback element, the small current signal could be converted to the large voltage with the conversion ratio of 10^9^ V/A. The conversion ratio is equal to the feedback resistance, and can be tuned easily. This result indicates that the printed organic OPAs can be used to measure a small current signal in the amperometric bio-sensors. The last demonstration is triangular and rectangular oscillators shown in Fig. 5d. The first part is composed of an organic OPA, four resistors and a capacitor, and generates a triangular wave. The second part is composed of another organic OPA  (or comparator), and generates a pulse-width-modulated (PWM) rectangular wave. The duty ratio of the PWM rectangular wave can be controlled by the DC voltage at *V*_IN_ terminal. Such PWM circuits can be used, e.g., for the printed wireless communication tags by connecting an analog sensor to the *V*_IN_ terminal.

### Conclusion

To conclude, we have developed the printed organic OPAs operatable at a standard and safe supply voltage of 5 V and demonstrated their versatility for a variety of signal processing. The metal electrodes and semiconductor layers were fabricated by an inkjet printer and a dispenser. The low voltage operation was realized by using dual-gate n-type OTFTs which exhibit a turn-on voltage close to zero, a small subthreshold slope *SS*, and a small channel length modulation coefficient *λ*. The versatility of the organic OPAs were demonstrated in voltage followers, non-inverting amplifiers, integrators, differentiators, current-to-voltage converters, and oscillators. It should be also possible to fabricate active filters, adders, subtractors, and digital-analog converters by employing the organic OPAs. We believe that the printed organic OPAs can play the most important role in disposable electronic patch sensing applications in near future.

## Methods

### Device fabrication

Complementary organic operational amplifiers (OPAs) were fabricated by combining dual-gate bottom-contact n-type organic thin-film transistors (OTFTs) and bottom-gate bottom-contact p-type OTFTs. After ultrasonic cleaning of the 700-µm-thick glass substrates in ultra-pure water, acetone and 2-propanol, a 300-nm-thick parylene dielectric layer (dix-SR, KISCO) was deposited onto the substrates in vacuum to modify the surface wettability (PDS 2010 LABOCOTER 2, Specialty Coating Systems, Inc.). Then, the bottom gate electrodes for n-type OTFTs were fabricated with an inkjet printer (DMP-2831, Fujifilm Dimatix Co.) and a silver nanoparticle ink (NPS-JL, Harima Chemical Inc.). The 10-pL cartridge and stage were kept at 40 °C and 45 °C, respectively, and the drop spacing was 60 µm. The silver ink was then sintered at 180 °C for 30 minutes in air. Then 246-nm-thick parylene gate dielectric layer was deposited in vacuum. After printing the silver electrodes and sintering at 180 °C for 30 minutes, a fluoropolymer solution (Teflon AF 1600, DuPont) in Fluorinert (FC-43, 3 M Co.) was patterned using a dispenser system (IMAGEMASTER 350, Musashi Engineering, Inc.) at 60 °C to control the dimensions of semiconductor layers, and dried at 100 °C for 30 minutes in nitrogen. The surface of source and drain electrodes for n-type OTFTs were modified by immersing the electrodes in a 10 mM solution of 4-methylbenzenethiol in 2-propanol for 5 minutes and then rinsing with 2-propanol. The n-type organic semiconductor layers were fabricated from a mixed solution of the 4,8-bis[5-(3-cyanophenyl)thiophene-2-yl]benzo[1,2-*c*:4,5-*c*’]bis[1,2,5]thiadiazole derivative (TU-3, Ube Industries, Ltd., Fig. 1a) at 0.06 wt% and poly(α-methylstyrene) (PαMS, average Mw ≈ 300,000, Sigma-Aldrich) at 0.02 wt% in 1-methylnaphthalene by a dispenser at 60 °C in air, and then annealed at 120 °C for 1 hour in nitrogen. Then, a 230-nm-thick parylene dielectric layer was deposited in vacuum. Inter-layer via holes were formed using an yttrium aluminum garnet (YAG) laser (VL-C30, V-Technology Co., Ltd.) with a wavelength of 355 nm. Then silver electrodes and fluoropolymer banks were fabricated in the same methods above. After immersing the electrodes in a 30 mM solution of pentafluorobenzenethiol in 2-propanol for 5 minutes and rinsing with pure 2-propanol, the p-type organic semiconductor films were finally deposited from a mixed solution of 2,8-difluoro-5,11-bis(triethylsilylethynyl)anthradithiophene (diF-TES-ADT) at 2 wt% and polystyrene (PS, average Mw ≈ 280,000, Sigma-Aldrich) at 0.5 wt% in mesitylene by inkjet printing. Finally, the devices were annealed at 120 °C for 1 hour in nitrogen.

### Device characterization

Electrical measurements were carried out in air. DC characteristics were measured using a semiconductor parameter analyzer (4200A-SCS, Keithley Instruments). AC and dynamic characteristics were measured using a waveform generator (AFG1022, Tektronix Inc.) and a digital oscilloscope (DSOS054A, Keysight Technologies Inc.). A voltage buffer (TLV2402, Texas Instruments Inc.) was inserted between the probe of the oscilloscope and the samples. Since the input bias current and the common-mode input capacitance of TLV2402 are as small as 100 pA and 3 pF, respectively, it can be a perfect output stage to drive a probe of the oscilloscope without affecting the OPA characteristics.

### Device simulation

Device simulations were carried out using LTspice XVII software. The device parameters for p-type and n-type OTFTs were as follows.

.model pOFET pmos(Level = 2 L = 45u W = 900u Uo = 0.07 Tox = 280n Vto = -1 Nfs = 3e11 Cgso = 1e-8 Cgdo = 1e-8 LAMBDA = 0.012)

.model nOFET nmos(Level = 2 L = 24u W = 800u Uo = 0.07 Tox = 280n Vto = 1 Nfs = 3e11 Cgso = 1e-8 Cgdo = 1e-8 LAMBDA = 0.005)

## Electronic supplementary material

Supplementary Information
