# Supplementary material for: Printed 5-V organic operational amplifiers for various signal processing
Source: Sci Rep. 2018 Jun 12;8:8980. doi: 10.1038/s41598-018-27205-7 (PMC5997680; doi:10.1038/s41598-018-27205-7)
Supplement: Supplementary file 1 — Supplementary Information [file 41598_2018_27205_MOESM1_ESM.pdf]

# Supplementary Information

## Printed 5-V organic operational amplifiers for various signal processing

Hiroyuki Matsui[1,*], Kazuma Hayasaka[1], Yasunori Takeda[1], Rei Shiwaku[1], Jimin Kwon[2], and Shizuo Tokito[1,*]

[1]Research Center for Organic Electronics (ROEL), Yamagata University, Yonezawa 992-8510, Japan
[2]Department of Creative IT Engineering, Pohang University of Science and Technology (POSTECH), Pohang 37673 , Korea

## 1. Variability of TFT and Operational Amplifier (OPA) Characteristics

In order to evaluate the variability of device characteristics, 10 devices for each device structure on a same substrate were measured. All of the transfer characteristics and parameters are shown in Figure S1 and Table S1.

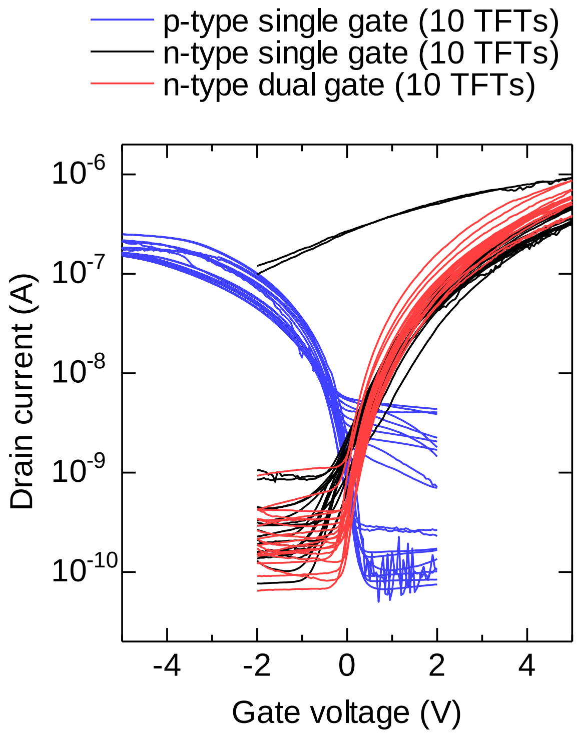


Fig. S1 Transfer characteristics of 10 TFTs for each structure. Drain voltage was +5 V for n-type and -5 V for p-type.

Table S1 Average and standard deviation of mobility and threshold voltage for the data in Fig. S1.

| | mobility ($cm^2/Vs$) | threshold voltage (V) |
|---|---|---|
| p-type single gate | 0.08 ± 0.01 | 0.35 ± 0.09 |
| n-type single gate | 0.06 ± 0.02 | 0.10 ± 0.07 |
| n-type dual gate | 0.04 ± 0.01 | 0.19 ± 0.06 |

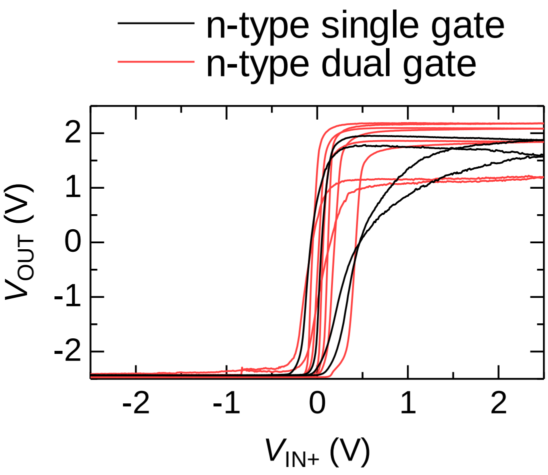


Fig. S2 Characteristics of 4 OPAs with dual-gate n-type TFTs and 2 OPAs with single-gate n-type TFTs. $V_{DD}$ = +2.5 V, $V_{SS}$ = -2.5 V, and $V_{IN-}$= 0 V.

## 2. Device Simulation in Finite Element Method (FEM)

Device simulation in finite element method (FEM) was carried out to investigate the carrier distribution in single- and dual-gate n-type transistors by using the mathematics module of COMSOL multiphysics (KESCO Ltd.). The simulation is based on the Poisson equation and the continuity equation.

$$\vec{\nabla} \cdot \left[\vec{\nabla} V(x,y)\right] = \frac{e}{\epsilon}\left[n_{\mathrm{f}}(x,y) + n_{\mathrm{t}}(x,y) - n_0\right]$$

$$\vec{\nabla} \cdot \left[e n_{\mathrm{f}}(x,y)\mu \vec{\nabla} E_{\mathrm{F}}(x,y)\right] = 0$$

Here $V$ is the electrostatic potential, $e$ is the elementary charge, $\varepsilon$ is the dielectric constant, $n_{\mathrm{f}}$ is the free electron carrier density, $n_{\mathrm{t}}$ is the trapped electron carrier density, $n_0$ is the carrier density in the neutral state, $\mu$ is the mobility tensor, and $E_{\mathrm{F}}$ is the Fermi energy. Carrier densities $n_{\mathrm{f}}$ and $n_{\mathrm{t}}$ were approximated as

$$n_{\mathrm{f}}(x,y) = D_{\mathrm{c}} \exp\left(\frac{E_{\mathrm{F}}(x,y) - E_{\mathrm{c}}}{k_B T}\right)$$

$$n_{\mathrm{t}}(x,y) = D_{\mathrm{t}} \exp\left(\frac{E_{\mathrm{F}}(x,y) - E_{\mathrm{c}}}{E_{\mathrm{t}}}\right)$$

Here $D_{\mathrm{c}}$ is the effective density of states in the conduction band, $E_{\mathrm{c}}$ is the bottom energy of the conduction band, $k_{\mathrm{B}}$ is the Boltzmann constant, $T$ is the temperature, $D_{\mathrm{t}}$ is the density of trap states, and $E_{\mathrm{t}}$ is the average energy of traps with exponential density of states. The parameters used in the simulation is listed in Table S2. Figure S3(b) displays the simulated transfer characteristics in the single- and dual-gate n-type transistors. The dual-gate transistor exhibited lower drain current at $V_{\mathrm{G}} < 1$ V than the single-gate transistors, which is consistent with the experimental results in Fig. 1b. Figure S3(c) and (d) show the distribution of free carrier density, $n_{\mathrm{f}}$, along the y axis in the single- and dual-gate transistors. It clearly indicates that the carrier density at the opposite side of the gate electrode is hardly controlled in the single gate transistor especially at $V_{\mathrm{G}} < 1$ V. By contrast, the dual gate can control the carrier density well over the entire region of the channel. This is why the dual-gate transistors exhibited smaller subthreshold slope, *SS*, than the single-gate transistors.

Table S2 Parameters used in the device simulation

| **Parameter** | **Value** | **Parameter** | **Value** |
|---|---|---|---|
| channel width | 1000 μm | workfunction of source and drain electrodes | 5.35 eV |
| channel length | 24 μm | workfunction of gate electrode | 6.0 eV |
| channel thickness | 200 nm | electron affinity | 5.1 eV |
| dielectric constant, $\varepsilon$ | $4\varepsilon_0$ | effective density of states, $D_c$ | $1.6 \times 10^{20}$ cm$^{-3}$ |
| thickness of gate insulator | 240 nm | density of trap states, $D_t$ | $5 \times 10^{16}$ cm$^{-3}$ |
| temperature, $T$ | 300 K | average energy of trap states, $E_t$ | 0.15 eV |
| mobility along the channel, $\mu_x$ | 0.07 cm$^2$/Vs | carrier density in the neutral state, $n_0$ | $2.6 \times 10^{12}$ cm$^{-3}$ |
| mobility perpendicular to the channel, $\mu_y$ | $7 \times 10^{-5}$ cm$^2$/Vs | | |

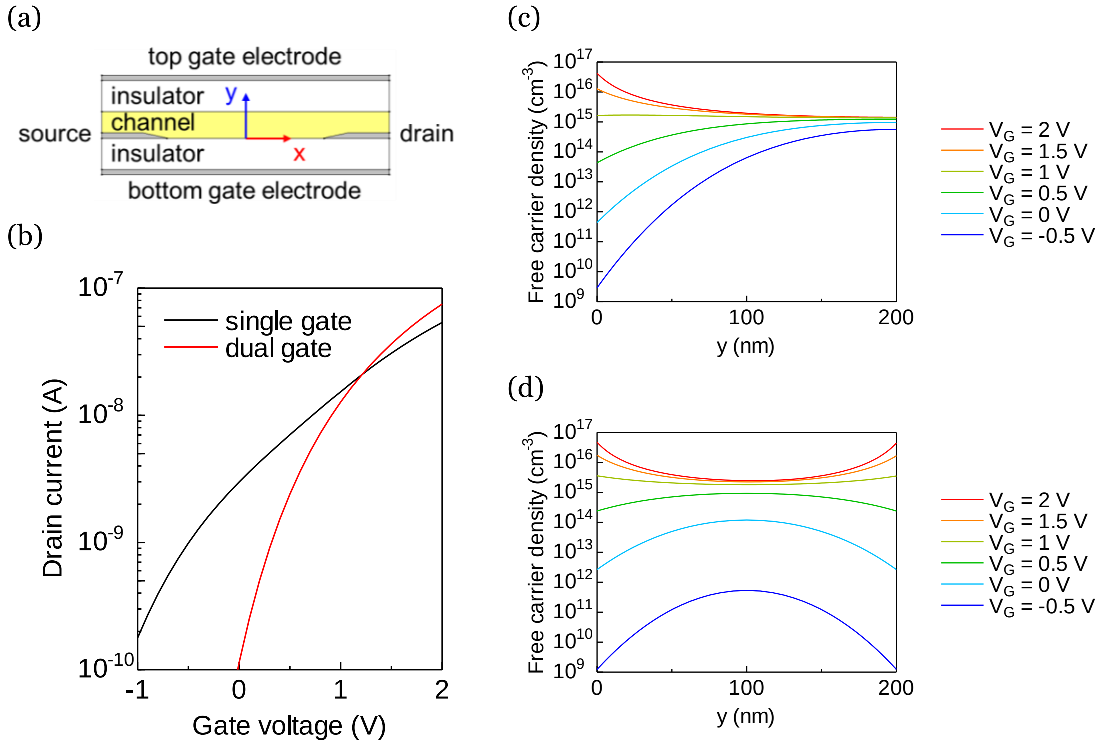


Fig. S3 Device simulation in finite element method (FEM). (a) Device geometry in the simulation. (b) Simulated transfer characteristics in saturation regime. (c) Free carrier density along y axis in the single gate and (d) dual gate n-type transistors.
